# Monitoring of controlled ovarian stimulation in IVF

**DOI:** 10.1007/s10815-024-03182-x

**Published:** 2024-07-04

**Authors:** Shahar Kol, Juan Carlos Castillo Farfan, Mark P. Trolice, Alexander M. Quaas

**Affiliations:** 1 IVF Unit, Elisha Hospital, Haifa, Israel; 2 Department of Reproductive Medicine, Instituto Bernabeu, Alicante, Spain; 3 Fertility Care, The IVF Center Orlando, Orlando, FL USA; 4 Shady Grove Fertility, 462 Stevens Ave., Ste. 206, Solana Beach, San Diego, CA 92075 USA

**Keywords:** In vitro fertilization (IVF), Assisted reproductive technology (ART), Controlled ovarian stimulation (COS), Ovarian stimulation protocols, Ultrasound monitoring in IVF

## Abstract

Since the inception of in vitro fertilization (IVF), monitoring of controlled ovarian stimulation (COS) has traditionally involved numerous appointments for ultrasound and laboratory testing to guide medication use and dosing, determine trigger timing, and allow for measures to reduce the risk of ovarian hyperstimulation syndrome (OHSS). Recent advances in the field of assisted reproductive technology (ART) have called into question the timing and frequency of COS monitoring appointments, as discussed in this commentary.

## Introduction

How frequently should we monitor patients undergoing controlled ovarian stimulation (COS) before in vitro fertilization (IVF)?

## The foundation of COS

It has been postulated that a target number of 11–18 oocytes retrieved in one stimulation represents a balance between efficacy and safety [1], although the cumulative live birth rate continues to improve with increasing oocyte numbers [2]. The gonadotropin starting dose is routinely determined by the individual patient’s ovarian reserve parameters, although it does not appear to influence the rates of live birth/ongoing pregnancy [3]. In addition, there is significant intra-individual variation in ovarian response between repeated cycles with the same ovarian stimulation protocol, perhaps influenced by waves of follicular recruitment into the antral follicular pool. This variability was not explained by baseline ovarian reserve parameters [4]. Recruitment waves do not appear to synchronize more with the traditional early follicular start of COS. Patients commonly expect a change in ovarian stimulation protocol after a previous failed attempt, although existing data on this topic do not support this approach [5]. Admittedly, despite all our efforts at optimizing COS, the outcome of a specific IVF cycle is partially determined by random events beyond our control, such as the synchronicity with antral follicular waves and the quality of the specific cohort of retrieved oocytes.

Protocols for clinical trials supported by the pharmaceutical industry frequently mandated that when using the antagonist protocol, the same stimulation dose is kept for 5 days, and then changed according to patient monitoring by blood tests and ultrasound scans [6–8]. However, recent advancements, such as GnRH agonist triggering, vitrification, freeze-all for genetic testing purposes, and progestin-primed ovarian stimulation (PPOS), are re-shaping COS protocols, offering new pathways and possibilities. While the value of estradiol monitoring during COS has already been questioned [9, 10], will inconvenient serial follicle measurements also be relegated to history? It seems intuitive that the monitoring schedule could be re-visited and adapted as well.

## Baseline ultrasounds, a fond memory?

The utility of the “baseline” scan in follicle monitoring is questionable when considering its alleged purpose: performing an antral follicle count (AFC) and identifying ovarian cysts that might justify postponement. From the standpoint of ovarian evaluation for IVF, the significance of conducting such scans at the commencement of COS becomes superfluous for multiple reasons. The AFC can vary throughout the menstrual cycle [11], and the “random start” ovarian stimulation protocol has been shown to yield an equivalent number of oocytes in various ART settings, including fertility preservation and oocyte donation [12, 13]. Furthermore, the use of the GnRH agonist trigger has nearly eradicated the risk of the heretofore bane of ovarian hyperstimulation syndrome (OHSS) [14, 15].

Most patients have undergone comprehensive assessments as part of their pre-ART work-up. Thus, the query arises: should we routinely conduct scans on the starting day of COS? Arguably not. This step could be viewed as optional rather than mandatory, with limited evidence suggesting a discernible association with improved outcomes. The consideration of whether such scans truly contribute significantly to the overall success of IVF prompts a reevaluation of their necessity in the protocol [16].

## When should we start ultrasound monitoring in COS?

If we follow the recommendation of using a fixed starting gonadotropin dose for at least 5–7 days [17], and since most patients are triggered on day 9 of stimulation [16], the conclusion is simple: same dose for 7 or even 8 days (depending on patient age: shorter follicular phase in advanced maternal age patients), and a single scan to determine the trigger day. Thus, only a single patient visit to the clinic is needed, during which blood hormones are measured, an ultrasound scan is performed, and in most cases, the trigger day is determined by the results. If the patient’s clinical characteristics and ovarian response indicate a high risk of OHSS, a GnRH agonist trigger should be used for a “freeze-all” embryo cryopreservation cycle, toward the goal of an OHSS-free clinic [18].

## Future directions

In recent years, we have witnessed a shift from fresh transfers to “freeze-all” cycles followed by frozen embryo transfer (FET). As we are becoming increasingly aware of the importance of a functional corpus luteum in FET cycles, we can follow the dominant follicle in a natural or modified-natural cycle to determine the ideal time for triggering and scheduling the FET. Arguably, close monitoring is more crucial in modified-natural FET cycles than during ovarian stimulation prior to egg retrieval. We propose that follicle monitoring needlessly burdens patients undergoing COS for IVF and that the currently high number of surveillance visits can safely be reduced to a much lower number, with possibly only one visit prior to egg retrieval. While the value of estradiol monitoring during COS has already been questioned [9, 10], it seems intuitive that the inconvenience of the traditional serial follicle monitoring schedule could be re-visited and, perhaps, relegated to history.
